# BRET Biosensor Analysis of Receptor Tyrosine Kinase Functionality

**DOI:** 10.3389/fendo.2013.00046

**Published:** 2013-04-09

**Authors:** Sana Siddiqui, Wei-Na Cong, Caitlin M. Daimon, Bronwen Martin, Stuart Maudsley

**Affiliations:** ^1^Receptor Pharmacology Unit, National Institute on Aging, National Institutes of HealthBaltimore, MD, USA; ^2^Metabolism Unit, National Institute on Aging, National Institutes of HealthBaltimore, MD, USA

**Keywords:** receptor tyrosine kinase, RTK, protein–protein interaction, neurotrophic, insulin receptor, insulin-like growth factor receptor, epidermal growth factor receptor, cytokine receptors

## Abstract

Bioluminescence resonance energy transfer (BRET) is an improved version of earlier resonance energy transfer technologies used for the analysis of biomolecular protein interaction. BRET analysis can be applied to many transmembrane receptor classes, however the majority of the early published literature on BRET has focused on G protein-coupled receptor (GPCR) research. In contrast, there is limited scientific literature using BRET to investigate receptor tyrosine kinase (RTK) activity. This limited investigation is surprising as RTKs often employ dimerization as a key factor in their activation, as well as being important therapeutic targets in medicine, especially in the cases of cancer, diabetes, neurodegenerative, and respiratory conditions. In this review, we consider an array of studies pertinent to RTKs and other non-GPCR receptor protein–protein signaling interactions; more specifically we discuss receptor-protein interactions involved in the transmission of signaling communication. We have provided an overview of functional BRET studies associated with the RTK superfamily involving: neurotrophic receptors [e.g., tropomyosin-related kinase (Trk) and p75 neurotrophin receptor (p75NTR)]; insulinotropic receptors [e.g., insulin receptor (IR) and insulin-like growth factor receptor (IGFR)] and growth factor receptors [e.g., ErbB receptors including the EGFR, the fibroblast growth factor receptor (FGFR), the vascular endothelial growth factor receptor (VEGFR) and the c-kit and platelet-derived growth factor receptor (PDGFR)]. In addition, we review BRET-mediated studies of other tyrosine kinase-associated receptors including cytokine receptors, i.e., leptin receptor (OB-R) and the growth hormone receptor (GHR). It is clear even from the relatively sparse experimental RTK BRET evidence that there is tremendous potential for this technological application for the functional investigation of RTK biology.

## Introduction

**Figure 1:**
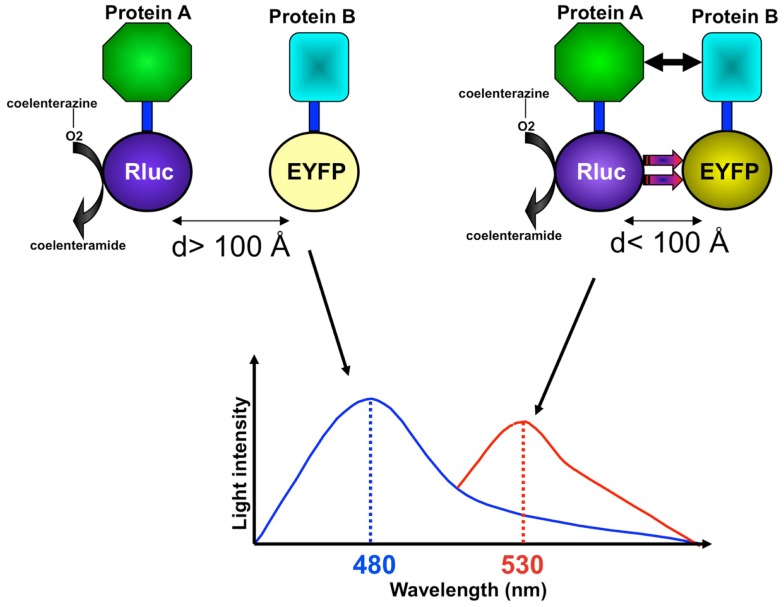
**The BRET assay has been developed to study protein–protein interactions**. In an example of studying the interaction between protein A and protein B using the BRET assay, fusion proteins with Rluc and YFP are coexpressed, and luminescent signals are measured at 480 nm (Rluc light emission) and 530 nm (YFP light emission) upon addition of the Rluc substrate coelenterazine. If protein A does not interact with protein B and if Rluc and YFP are not at a BRET-permissive distance (>100 Å) and orientation, non-radioactive light emission is mainly measured at 480 nm. If protein A is in close proximity, or interacts with, protein B, placing Rluc and YFP at a BRET-permissive distance (<100 Å) and orientation, non-radioactive energy transfer can be measured at an increased light emission at 530 nm.

As a natural phenomenon, bioluminescence is found in marine animals such as the sea pansy *Renilla reniformis* and the jellyfish *Aequorea victoria*. Research has demonstrated that the oxidation of the intrinsically produced substrate coelenterazine to coelenteramide initializes the bioluminescence in those organisms (Figure 1) (Hart et al., 1978; Pfleger and Eidne, 2003). Bioluminescence resonance energy transfer (BRET) simply represents an energy transfer from a luminescent donor to a fluorescent acceptor, which re-emits light at another wavelength. BRET requires a sufficient overlap between the emission spectrum of a donor molecule and the absorption spectrum of an acceptor molecule (Figure 1) (Issad et al., 2002). BRET also depends on the distance between the donor and the acceptor, which should be in the range of 10–100 Å, and on their interacting orientation (Figure 1) (Wu and Brand, 1994). Based on this principle, the BRET assay has been developed and applied to study protein–protein interactions as a facile methodological tool.

An important advantage of the BRET assay is that it allows researchers to study dynamic protein–protein interactions in living cells (Hamdan et al., 2006). In general, BRET assays involve proteins of interest fused with either a donor molecule (Renilla luciferase, or Rluc) or an acceptor molecule [usually a variant of green fluorescent protein (GFP)/enhanced yellow fluorescent protein (EYFP)]. BRET fusion proteins are created by expressing specifically engineered cDNAs from both the protein of interest and the donor or acceptor molecule. Subsequently, both donor-tagged and acceptor-tagged constructs are co-transfected into host cells. The presence of energy transfer between the donor and acceptor molecules can then be measured. The amount of energy transference correlates with the extent to which the specific tagged molecules exist within proximity of each other. The wavelengths for detection differ according to the use of BRET (480 nm for Rluc and 530 nm for EYFP) (Figure 1). The original BRET technology generally used EYFP as an acceptor, a red-shifted variant of YFP that has an emission maximum at 530 nm. In contrast, the recently introduced BRET-2 uses a codon of humanized wild-type GFP form, termed GFP2. GFP2 has a maximal emission at 510 nm. The BRET-2 system is designed to increase the spectral resolution compared to the original BRET technology. The improved resolution is attributed to the application of DeepBlue C coelenterazine with Rluc and GFP2, resulting in better separation of the luciferase/DeepBlue and GFP2 emission peaks. Whereas, in the original BRET technology, the h form of coelenterazine with Rluc and EYFP is used (Pfleger and Eidne, 2003). However, one of the limitations of BRET-2 is its lower efficiency of light emission that implies overexpression of the partners at supraphysiological levels.

Unlike fluorescence resonance energy transfer (FRET), BRET-based systems do not require the excitation of the donor with an external light source thus, minimizing the unnecessary autofluorescence, light scattering, photobleaching, and the possible photoisomerization of the donor, or even photodamage to the cells. BRET also allows detection of smaller variations in BRET signals as there is low background in the BRET assays due to the absence of any contamination of the light output. Ratiometric measurements of BRET minimize any variations that may occur due to a wide variety of possibilities including: differences in assay volumes, cell types, and numbers, as well as a decay of a signal in a given plate. As with other bioluminescence-based assays, BRET performance can be significantly affected by several factors, including the spectral properties of donor and acceptor molecules (Xu et al., 1999), the ratio of donor to acceptor molecules (Gomes et al., 2002), the distance and orientation of the molecules of interest (Wu and Brand, 1994; Kenworthy, 2001) and the strength and stability of the interactions (Pfleger and Eidne, 2003). Therefore, while presenting multiple advances over previous technologies such as FRET, BRET-based approaches can have their functional limitations. Using BRET to study protein–protein interactions may be critiqued for providing a potentially skewed view of biomolecular interactions. Biomolecular complexes are likely to contain tens or even hundreds of proteins at times and due to the relatively limited number of BRET probes, the number of simultaneous interactions that can be monitored is worryingly limited. As BRET employs ectopically expressed factors there is also an issue of both the lack of endogenous regulation of expression, cellular disposition, and compartmentalization of the factor. Expressing a novel factor in a cell line is highly likely to disrupt the stoichiometry of multiple signaling systems with potentially unknown consequences (Martin et al., 2009a). In addition to this, the variable nature of the host-cell environment, e.g., passage number, differentiation methodologies or viral transformation, will also likely affect signaling systems investigated using ectopically expressed BRET probes. Ideally, molecular interactions should be studied with native-state proteins as the addition of BRET labels may also affect the physico-chemical properties of the protein which may change its transport between different cell compartments, its post-translational modification status, its protein–protein interactions, and even its degradative processing. Changes to any of these properties of the target protein will likely have a significant impact on its perceived functionality using the BRET technique.

The BRET assay was first described in a study on the dimerization of the bacterial Kai B clock protein (Xu et al., 1999). Prior to this first BRET demonstration, non-BRET bioluminescent technologies were employed by Barak et al. (1997) to investigate the functional signaling activity of G protein-coupled receptors via β-arrestin-GFP translocation to the plasma membrane. Following this, a considerable body of BRET-based G protein-coupled receptor (GPCR) functional analysis has now been generated (Angers et al., 2000; Galés et al., 2005; Ayoub et al., 2007). In addition, the activation or inactivation of second messengers such as cyclic adenosine monophosphate (cAMP) generated by GPCR activation, has also been well-studied using BRET. These techniques include the fusion of the regulatory and catalytic subunits of protein kinase A (PKA) to GFP and Rluc biosensors in order to monitor cAMP activity (Prinz et al., 2006), or the fusion of biosensors to the guanine nucleotide exchange protein activated by cAMP (Jiang et al., 2007; Barak et al., 2008). While BRET has been exhaustively employed for GPCR-based studies, in this present review, we instead focus on the applications of the BRET assays in the functional investigations of the receptor tyrosine kinase (RTK) superfamily. This superfamily contains a variety of distinct receptors associated with diverse functional activities. Hence, the RTK superfamily includes neurotrophic receptors such as tropomyosin-related kinase (Trk) and p75 neurotrophin receptor (p75NTR), insulinotropic receptors including the insulin receptor (IR) and insulin-like growth factor receptor (IGFR), as well as growth factor receptors such as the ErbB receptors including the epidermal growth factor receptor (EGFR), the fibroblast growth factor receptor (FGFR), the vascular endothelial growth factor receptor (VEGFR), and the c-kit and platelet-derived growth factor receptor (PDGFR). Cytokine receptors, e.g., leptin and growth hormone receptors (GHR), while not being traditional RTKs, possess multiple functional similarities with RTKs, e.g., receptor dimerization tyrosine kinase usage, and as such have also been investigated with BRET-based approaches.

## Investigating GPCR Signaling with BRET

Bioluminescence resonance energy transfer approaches have been extensively applied to the investigation of the dimerization or other protein–protein interactions of multiple types of GPCRs, e.g., melatonin receptors (Ayoub et al., 2002), chemokine receptors (CXCR1, 2, and 4 and CCR2 and 5) (Milligan et al., 2005), α/β-adrenergic receptors (Angers et al., 2000; Small et al., 2006), cholecystokinin receptors (Harikumar et al., 2006), yeast α-factor receptors (Gehret et al., 2006), opsin receptors (Vrecl et al., 2006), protease-activated receptor 1 (Ayoub et al., 2012), and secretin receptors (Lisenbee and Miller, 2006). BRET has also been used to study the ability of muscarinic acetylcholine receptors, M3 and M5, to form homo- and hetero-dimers in living cells in a manner independent of receptor activation (Borroto-Escuela et al., 2010). As mentioned previously, one of the earliest BRET studies was used to assess whether the human β_2_-adrenergic receptor (β_2_AR), existed as a homodimer in living cells (Angers et al., 2000). This study found that GPCRs exist as functional dimers in the *in vivo* setting and therefore, BRET-based assays could be applied for the study of both constitutive and hormone-promoted selective protein–protein interactions (Angers et al., 2000). In addition to GPCR–GPCR interactions, both membrane and cytosolic protein interaction with GPCRs have been studied with BRET (Milligan, 2004; Pfleger and Eidne, 2005; Pfleger et al., 2006). For example, BRET1-based β-arrestin 2 translocation assays have been used to quantify receptor activation/inhibition (Hamdan et al., 2005). The BRET1 experimental approach is commonly used when it is important to maintain a systemic physiological protein expression level (Bacart et al., 2008). One pertinent study describes a BRET1-β-arrestin recruitment assay in stable mammalian cells and its successful application in high-throughput screening for GPCR antagonists (Hamdan et al., 2005).

## Investigating Tyrosine Kinase-Based Receptor Systems with BRET

While GPCRs form perhaps the most important pharmacotherapeutic target for drug research (Maudsley et al., 2005) it is still crucial to generate a diversity of therapeutic strategies to contend with disease pathophysiologies. Therefore, the development of RTK-based drug discovery is vital to support the already mature field of GPCR-based drug design. In addition to the important use of BRET-based techniques for GPCR research, BRET has also proven to be useful in monitoring RTK receptor functionality and assisting in drug discovery efforts for identifying novel RTK modulators (Tan et al., 2007). BRET has also been used to study the nature of the ligand-induced conformational changes that accompany signal transduction pathway activation in RTKs (Boute et al., 2001).

Receptor tyrosine kinases are a varied group of transmembrane proteins acting as receptors for cytokines, growth factors, hormones, and other signaling molecules. RTKs are expressed in many cell types and play important roles in a wide variety of cellular processes, including growth, differentiation, and angiogenesis. Many RTKs, characterized by the archetypical EGFR, are composed of a single transmembrane helical region, a large extracellular immunoglobulin-like N-terminal domain and an intracellular C-terminal domain possessing an intrinsic tyrosine kinase activity. Cytokine receptors, while not possessing an intrinsic tyrosine kinase activity in their C-terminal domain, do actively recruit Janus kinase (Jak) family tyrosine kinase molecules to their intracellular domain to effect downstream signal transduction. Receptor dimerization, either ligand-driven or constitutive, forms an important component of the activation process of RTKs. These phenomena, therefore, make the investigation of their functionality with BRET highly analogous to the use of BRET in GPCR studies. Ligand-mediated RTK dimerization, e.g., for EGFR or PDGFR, or constitutive dimerization, e.g., for insulin/insulin-like growth factor-1 receptor, results in the stimulation of either tyrosine kinase recruitment (Jak2) or activation of intrinsic tyrosine kinase activity (EGFR). These active tyrosine kinases can then phosphorylate downstream signaling molecules as well as the opposing dimer unit of the RTK (auto-tyrosine phosphorylation). These auto-tyrosine phosphorylation sites conform to the C-terminal domain of the RTK into a series of high-affinity binding sites for downstream signaling proteins which possess canonical Src-homology 2 (SH2) or protein phosphotyrosine binding (PTB) motifs. The assembly of multiple proteins with the C-terminal domain of the RTKs then serves to propagate and “*condition*” the downstream signaling of the receptor (Maudsley et al., 2000b; Martin et al., 2009a). A significant advancement in the appreciation of functional transmembrane receptor systems was made by Maudsley et al. (2000a,b) through their demonstration of the creation of “higher-order” multi-protein signaling entities between active GPCRs and RTKs. The discovery that GPCR-based signals can then merge and also condition RTK-mediated signaling has since been developed into an important field of research into the nature of receptor signaling transfer for many receptor systems (Gschwind et al., 2001; Sabri et al., 2002; Piiper et al., 2003; Sales et al., 2004; Flajolet et al., 2008; Chadwick et al., 2011a). This productive interaction therefore opens up the potentially important application of BRET-based techniques for the investigation of this emerging paradigm in receptor biology. Eventually it is likely that with BRET-mediated high-content screening (HCS) techniques, receptor ligands possessing a predilection for activating this RTK-associated GPCR “ensemble” may be rationally discovered and therefore constitute a novel and unique pharmacological resource (Maudsley et al., 2005). In the following sections of this review, we will discuss the most recently developed experimental evidence and concepts derived from RTK-associated BRET research. Each of the target receptor systems is likely to represent some of the most important future therapeutic targets, given the need for increased diversity in therapeutic mechanisms for the future pharmacopeia.

### BRET for labeling of neurotrophic receptors

The neurotrophins are a family of closely related signaling proteins that control a number of crucial aspects of neuronal (both central and peripheral) activity, i.e., survival, development, responses to stress, and synaptic reinforcement (Mattson et al., 2004a; Skaper, 2008; Stranahan et al., 2009; Golden et al., 2010; Chadwick et al., 2011b; Driscoll et al., 2012). In mammals, the Trk subfamily of RTKs constitutes one major class of neurotrophic tyrosine kinase receptors. Sharing the typical features of RTKs, the activation of Trk receptors is often triggered by neurotrophin-mediated dimerization and/or transphosphorylation of an activation loop kinase (Huang and Reichardt, 2003). Most mammalian neurotrophins elicit their biological functions by activating one or more of the three members of the Trk family of RTKs (TrkA, TrkB, and TrkC) (Kaplan et al., 1991; Klein et al., 1991; Lamballe et al., 1991; Chadwick et al., 2010; Park et al., 2011). Being able to accurately monitor Trk activities in living cells will likely provide a platform for both drug development and mechanism-based research.

Based on the original BRET technology, Tan et al. (2007) further developed BRET-2 assays specifically for evaluating the interactions between Trk receptors (TrkA, TrkB, TrkC) and three kinds of effectors (p85, Shc46, phospholipase C gamma, PLCγ1) with three different neurotrophic stimulators (nerve growth factor, NGF, brain-derived neurotrophic factor, BDNF, neurotrophin-3, NT-3). To briefly describe the BRET-2 process, the size of the BRET-2 signal is expressed as the ratio of GFP2 and luciferase emissions, which correlates with the extent of recruitment of the effector proteins to the Trks, once Trks are activated. Under the stimulation of agonists including NGF (TrkA), BDNF (TrkB), and NT-3 (TrkC), interactions of TrkA-p85/Shc46/PLCγ1, TrkB-p85/Shc46/PLCγ1, and TrkC-Shc46 were continuously monitored, generating both BRET-2 ratio/log [concentration] curves as well as the EC_50_ for each ligand. Similarly, under the inhibition with the antagonist K252a, the same recruiting interactions were also captured, generating IC_50_ values, as well. All together, using BRET-2, this group successfully demonstrated that multiple forms of Trk activity can be investigated in live cells and may represent a reliable core technology for evaluating Trk activity and responsiveness to novel therapeutics.

The BRET assay-based monitoring system has also been used to answer several conformational and mechanistic questions related to functions of Trk receptors. Overexpression of TrkB has been linked to neuroblastomas (Brodeur, 2003) as well as other types of cancers (Moon et al., 2011; Fujikawa et al., 2012). TrkB kinase activity has also been shown to be responsible for the induction of metastasis by the suppression of anoikis, a form of apoptosis due to incorrect or inadequate cell and extracellular matrix attachment (Douma et al., 2004). Additionally, a growing body of evidence demonstrates that TrkB-mediated BDNF signaling plays a critical role in the pathogenesis of multiple neurodegenerative disorders such Alzheimer’s disease (AD) and Huntington’s disease (Martin et al., 2009b, 2012; Chadwick et al., 2011b; Cong et al., 2012). With the application of the BRET assay, De Vries et al. (2010) demonstrated a conformational rearrangement of preformed TrkB/Shc complexes initialized by BDNF-dependent activation, revealing a complex level of interaction between TrkB and Shc. It is noteworthy that in the study by De Vries et al. (2010), both TrkB receptor mutants as well as compound blockers were tested with the BRET assay. Therefore again, this further suggests that the TrkB BRET assay could be utilized to investigate Trk signaling and potential therapeutic design and provides a good example for the BRET assay application in labeling neurotrophic receptors. This study highlights the application of the BRET saturation assay which allows the determination of a conformational rearrangement of preformed complexes versus the recruitment of one signaling molecule to another, the latter being indicative of the relative affinity of two interacting molecules. This application has also been highlighted in earlier studies (Lacasa et al., 2005; Nouaille et al., 2006).

The p75NTR, a C-terminally truncated, non-signaling Trk receptor modulator (Segal, 2003; Makkerh et al., 2005) is involved in the regulation of multiple neuronal activities, e.g., development of neurodevelopmental processes (Nykjaer et al., 2005), neuronal migration (Johnston et al., 2007; Snapyan et al., 2009), and also neuronal growth inhibition (Yamashita et al., 1999; von Schack et al., 2001). Physically p75NTR can potentiate Trk signaling by potentiating neurotrophin ligand binding to TrkA receptors (Barker and Shooter, 1994; Hantzopoulos et al., 1994) thus enhancing cellular neurotrophin sensitivity (Yamashita et al., 1999; von Schack et al., 2001; Ito et al., 2003). The BRET assay has also been used for studying the interactions between the amyloid precursor protein, that is strongly implicated in AD pathophysiology, and p75NTR (Fombonne et al., 2009). Based on the BRET results, the connection between amyloid precursor protein and p75NTR is one of the most selective interactions observed in AD.

### BRET assay for labeling insulinotropic receptors

Insulin, a complex peptide hormone secreted by the beta cells of the Islets of Langerhans in the pancreas, controls energy metabolism in the liver, muscle, and adipose tissue by binding to its cognate transmembrane tyrosine kinase receptor, i.e., the IR. Alterations in insulin signaling and action lead to pathophysiological conditions such as obesity, Type 2 diabetes mellitus (T2DM), and generalized metabolic syndrome (Maudsley et al., 2011). The IR is composed of two extracellular alpha-chains that bind ligands and two transmembrane and intracellular β-subunit chains that possess the tyrosine kinase activity. The IR can be considered to be a “pre-dimerized” analog of growth factor receptors such as the EGFR. While the IR is effectively dimerized before the interaction with the peptide ligand, binding of insulin induces a conformational change that allows transphosphorylation of one β-subunit of the IR by the ligand-mediated stimulation of the intrinsic tyrosine kinase activity of the other β-subunit. BRET assays are highly sensitive for quantifying ligand-independent (constitutive), agonist-induced or antagonist-inhibited RTK activity levels (Tan et al., 2007). The first use of BRET to quantify constitutive, agonist-induced and antagonist-induced RTK activity was performed by Boute et al. (2001), using hormones, growth factors, as well as monoclonal antibodies (Boute et al., 2001). Blanquart et al. (2008) have utilized BRET to characterize ligand-induced conformational changes that occur within hybrids of IRA/IRB, the two isoforms of IR either containing or not containing exon 11 (Blanquart et al., 2008). IRA/IRB hybrids have been reported to be produced randomly in cells (Blanquart et al., 2008).

The discovery of pharmacological agents that specifically activate the tyrosine kinase activity of the IR will be of great importance for the treatment of insulin-resistant or insulin-deficient patients. As functional homologs to insulin, the insulin-like growth factors (IGF-I and IGF-II) play important roles in regulating growth, development, and differentiation of cells (Dupont and LeRoith, 2001) by binding to their cognate IGF-I receptor (IGF-1R). Similar to the IR, IGF-1R also belongs to the RTK superfamily (De Meyts and Whittaker, 2002). IGFRs are widely expressed throughout the central nervous system (CNS) as well as in the majority of peripheral tissues. BRET has facilitated the detection of the activation state of the IGF-1R, independently of any phosphorylation event by allowing the measurement of structural changes to the receptor in response to its cognate ligand (Blanquart et al., 2005). Activation of IGFR has been strongly implicated in generating a protective mechanism favoring neuronal cell survival and regeneration, which makes IGFR a potential therapeutic target for treating brain ischemic injury and neurodegenerative disorders (Roudabush et al., 2000; Mattson et al., 2004b; Harvie et al., 2011; Zemva and Schubert, 2011).

In order to evaluate the activity of IR and IGFR signaling pathways, both rapidly and in real-time, different BRET assays have been optimized for multiple applications. BRET assays for the real-time monitoring of the IR activity in living cells have been applied to investigate the molecular nature of binding partner interactions [growth factor receptor-bound protein 14, Grb14 (Nouaille et al., 2006)], the identification of novel IR system interactors [e.g., Sam68 (Quintana-Portillo et al., 2012)], as well as the activation mechanism of the IRs themselves (Boute et al., 2001). Furthermore, the BRET assay can also be applied to demonstrate or verify poor interactions between the IR and its substrates. IR substrates (IRS)-5 and -6 are two recently identified members of the IRS family. With the application of the BRET assay, Versteyhe et al. (2010) illustrated the finding that IRS-5 and IRS-6 are poor substrates for the IR compared to IRS1 and Shc (Versteyhe et al., 2010). More recently, using the BRET-2 assay in IR-Rluc8 and IRS(1,4,5)/Shc-GFP2 co-transfected HEK293 cells, Kulahin et al. (2012) examined interactions between IR and the canonical IRS (IRS1, IRS4) as well as the bifunctional SH2-domain-containing adaptor protein Shc. With this experimental paradigm, this group was able to demonstrate that specific insulin analogs may possess a 10-fold more potent capacity for the recruitment of IRS1, IRS4, and Shc, compared to human insulin. These varied studies suggest that the IR-based BRET assay may be a valuable tool to discover molecules with insulin-like properties.

Blanquart et al. (2005, 2006) have also applied BRET assays to pursue mechanistic questions into greater depth concerning the conformational changes of IGFR or IR induced by negative regulators such as PTP1B. Earlier, Boute et al. (2003) described the monitoring of the interactional dynamics of IR with PTP1B upon insulin stimulation. In 2005, using BRET, it was demonstrated that with insulin stimulation, the interaction of IR with receptor-like protein tyrosine phosphatases (PTPalpha and PTPepsilon) was due to conformational changes within preassociated IR/protein tyrosine phosphatase complexes (Lacasa et al., 2005). Later in 2011, Boubekeur et al. (2011) showed the interaction of PTP1B with the IR precursor during its biosynthesis in the endoplasmic reticulum. Similar to the IR-based BRET assay, co-transfection of Rluc or YFP-fused IGFR in HEK293 cells constitutes the ligand-induced conformation monitoring BRET assay. Additionally, by co-transfecting both IGF-1R-Rluc and YFP-PTP1B in HEK293 cells, the researchers were able to further reveal the interactions between IGF-1R and the negative regulator PTP1B in response to IGF1, IGF2, or insulin. Taken together from these varied studies, BRET assays are a useful technique for studying ligand-induced IR/IGFR conformational changes, assessing interactions between IR/IGFR and their negative or positive cellular partners or modulators, and setting up the platform of high-throughput screening for leading compounds relevant to related disorders.

Recently, in 2012, BRET was used to study the effects of insulin analogs on IR/IGF-1R hybrids. The group reported that when using MCF-7 cells (human breast adenocarcinoma cell line), glargine, which possibly acts via IR/IFG1R hybrids, demonstrated higher potency while its metabolites, M1 and M2, display lower potency than insulin for the stimulation of proliferative/anti-apoptotic pathways (Pierre-Eugene et al., 2012). They further developed a highly sensitive BRET-based assay that would allow monitoring of the production of phosphatidylinositol-3 phosphate (PIP3) upon stimulation of endogenous IR and IGF-1R in living cells (Pierre-Eugene et al., 2012).

### BRET labeling of growth factor receptors

Bioluminescence resonance energy transfer-based techniques can be used to either measure direct EGFR dimerization or to assess the binding of downstream signaling factors to the activated state of the receptor. BRET assays for EGFR have proven to be a useful tool to study the effective pharmacology of ligand-induced interaction between EGFR and signaling pathway-specifying adaptor proteins (Schiffer et al., 2007). Probing these interactions is crucial as EGFR has been classified to have a central role beyond cancer research in neurometabolic aging (Siddiqui et al., 2012) and conditions such as asthma, where EGFR has been shown to be upregulated in asthmatics (Amishima et al., 1998; Puddicombe et al., 2000), and chronic obstructive lung disease (COPD) where there is abundant mucus production, in which EGFR is known to play a role (Takeyama et al., 1999). *In vivo* rodent models confirm the importance of EGFR in asthma (Vargaftig and Singer, 2003; Tamaoka et al., 2008; Le Cras et al., 2011). The structural nature of the cognate ligand for EGFRs can also profoundly affect EGFR signaling. EGFR activation by stimulants such as histamine (Hirota et al., 2012), which does not classify with the commonly known axis of EGFR ligands, can also be assessed using BRET. Somatic mutations in epidermal growth factor (EGF) can produce ligand variants that quantitatively differ in their pharmacological and downstream signaling properties. This variability suggests the possibility of differential clinical responsiveness to treatment with EGFR inhibitors (Divgi et al., 1991; Perez-Soler et al., 1994; Modjtahedi et al., 1996; Baselga et al., 2000; Robert et al., 2001; Woodruff et al., 2010). EGFR is amongst other RTKs being probed as potential drug targets for asthma (Siddiqui et al., 2013).

In a profound BRET-facilitated study by Tan et al. (2007), the EGFR was shown to interact with Grb2 (growth factor receptor-binding protein 2) as well as Shc46 (MAP kinase proliferation pathway), PI3K-p85 regulatory subunit (PI3K-Akt survival pathway), PLCγ1 (protein kinase C/calcium signaling pathway), and STAT5a (from the signal transducers and activators of the transcription pathway) upon stimulation with the EGF. The ErbB4 growth factor receptor has also been shown to interact with Grb2 and p85 upon stimulation with one of the various ligands able to stimulate this receptor, i.e., heregulin-beta 1 (HRG-β1) (Tan et al., 2007). PDGFR A and B interacted with Grb2 and PLCγ1 when platelet-derived growth factor-BB (PDFG-BB) was used as a stimulant, while PDGFRA also interacted with p85 (Tan et al., 2007). Employing stem cell factor (SCF)-mediated activation of the c-Kit RTK, c-Kit was shown to dynamically interact with both Grb2 and p85 (Tan et al., 2007). Furthermore, vascular endothelial growth factor-C (VEGF-C) stimulation resulted in VEGFR3 and Grb2 interaction (Tan et al., 2007).

Fibroblast growth factor receptor and Grb14 intercommunication has also been investigated with BRET (Browaeys-Poly et al., 2010). Grb14 was found to bind to the phosphorylated FGFR where it induces a conformational change, and thereby unmasks a PLCγ-binding motif on Grb14, resulting in the inactivation of PLCγ (Browaeys-Poly et al., 2010). Therefore, using BRET analysis the authors of this study demonstrated their ability to measure the dynamic capacity of Grb14 to functionally inhibit FGFR signaling. In 2011, BRET was also used to assess the likelihood of FGFR1 homodimer formation upon stimulation by various FGF agonist ligands in HEK293T cells (Romero-Fernandez et al., 2011). FGFR1 is activated by homodimerization when FGF agonist ligand and heparin sulfate glycosaminoglycan are both present.

### BRET labeling of cytokine receptors

Activation of cytokine receptors by their cognate ligands induces a rapid recruitment of the Janus family of tyrosine kinases (Jak1/Jak2) in a Fyn- (Src-family tyrosine kinase) dependent manner. In the case of cytokine receptors (e.g., growth hormone, leptin, prolactin, or interleukin) the recruitment of the Jak kinases substitutes for the lack of an intrinsic tyrosine kinase activity in the C-terminal domain of these receptors. Hence, the ligand-induced association of Jak kinases with cytokine receptors in part recapitulates the functional signaling behavior of EGFR-like growth factor receptors. However, a specific function of the Jak recruitment is their ability to tyrosine phosphorylate downstream activators of transcription from the STAT family of proteins. The Jaks phosphorylate the intracellular tyrosines of the receptor complex, creating docking sites for STATs, which themselves become tyrosine-phosphorylated, thereby forming homo- or hetero-dimeric complexes that translocate to the nucleus. In the nucleus, STATs bind to specific gene promoters to activate the transcription of a range of targeted genes. In addition, autoinhibitory *Socs* (silencers of cytokine signaling) genes are also activated by cytokine receptor signaling via this Jak–STAT pathway (Starr et al., 1997). An assay-based on BRET was developed to detect the dimerization and action of the leptin receptor (OB-R), a type I cytokine receptor (Couturier and Jockers, 2003).

The short form of the prolactin receptor inhibits prolactin-induced activation of gene transcription by the long form of the prolactin receptor. In 2009, it was demonstrated using BRET that there is a higher homodimerization affinity of the mutated form of the short form of the prolactin receptor, reduced heterodimerization associations, long form homodimerization, and subsequent prolactin-induced signaling (Xie et al., 2009). Recently, a new genetically encoded biosensor based on BRET technology has been developed to allow real-time monitoring of inflammasome activity (Compan et al., 2012). The primary functional features of this sensor are similar to the endogenous IL-1β, which makes this probe an ideal tool for the characterization of pro-IL-1β processing and for the high-throughput screening of compounds that may underpin the initiation of inflammation (Compan et al., 2012).

Bioluminescence resonance energy transfer has also been successfully applied for the study of GHR activation (Brown et al., 2005). Along with FRET and co-immunoprecipitation in this particular study, BRET studies have generated important evidence that GHR subunits undergo specific transmembrane interactions independent of hormone binding (Brown et al., 2005).

## Use of BRET for the Study of RTK-Interacting Proteins

In addition to investigating receptor-specific RTK events, BRET can also be used to monitor RTK accessory protein binding. As briefly discussed earlier, currently 22 BRET assays for 9 RTKs, derived from 4 subfamilies [erythroblastic leukemia viral (v-erb-b) oncogene homolog (ErbB), PDGF, neurotrophic Trk, VEGF] have been reported that allow real-time monitoring of interactions with multiple effectors, i.e., Grb2, p85, Sta5a, Shc46, PLC-γ1 (Tan et al., 2007). Demonstrating BRETs utility in this field, BRET studies helped identify tyrosine residues 1068, 1114, 1148 as the main residues mediating interaction of EGFR with Grb2 (Tan et al., 2007). The use of BRET has also proven to be useful in understanding the often complex relationships between ligand-mediated RTK activation and sensitivity to chemical inhibitors of their function. BRET assays have thus suggested that the conformational rearrangement of preformed TrkB-Shc complexes, following BDNF-dependent activation, may prove extremely useful for the HCS of potential pharmacological blockers of TrkB signaling in a physiologically relevant context (De Vries et al., 2010).

Furthermore, BRET has also been used to study how alpha (v) beta (3) [α(v)β(3)] integrins cooperate with transmembrane receptor systems, such as tyrosine kinases, to enhance cellular responses (Scaffidi et al., 2004). Integrins are single-pass transmembrane receptors for extracellular matrix proteins such as fibronectin. While integrins themselves do not possess intrinsic tyrosine kinase activity, upon interaction with their extracellular matrix “ligand” molecule, they rapidly associate with tyrosine kinase scaffolding proteins such as focal adhesion kinase (FAK) and proline-rich tyrosine kinase 2 (Pyk2) (Della Rocca et al., 1999; Davidson et al., 2004; Maudsley et al., 2006, 2007). These scaffolding proteins, in a similar manner to the intrinsic tyrosine kinase domains of growth factor receptors such as the EGFR, upon interaction with integrin molecules activate their tyrosine kinase catalytic function. Once this activity is stimulated, these scaffolding proteins then undergo auto-tyrosine phosphorylation to create signaling protein docking sites. Therefore integrin receptors, as with cytokine receptors, replicate a form of classical RTK activity.

## Conclusion and Perspectives

**Figure 2:**
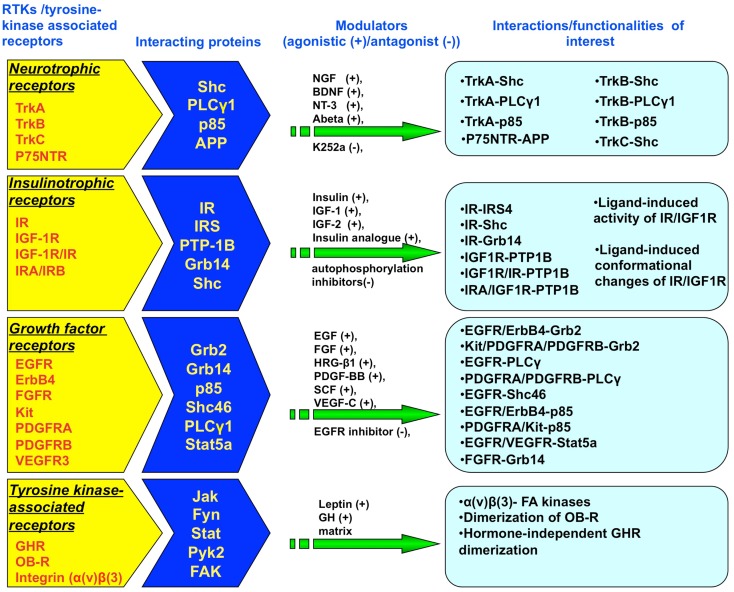
**Studies assessing protein–protein interactions for RTK and tyrosine kinase-associated receptors using Bioluminescence Resonance Energy Transfer (BRET)**. BRET assays have been established to study both the association of multiple RTK/tyrosine kinase-interacting proteins with the receptor superstructure as well as stimulator/inhibitor-mediated conformational changes in receptor structure. The RTK receptors include neurotrophic receptors (TrkA, TrkB, TrkC, p75NTR), insulinotropic receptors (IR, IGF-1R, IR-IGF-1R hybrid receptors), and growth factor receptors (FGFR, EGFR, ErbB4, kit, PDGFRA/B, VEGFR3). Tyrosine kinase-associated receptors include GHR, OB-R, and integrin receptors.

Bioluminescence resonance energy transfer is an advanced technology that can be applied in live cells and has been successfully applied to the investigation of protein–protein interactions, structure-function analysis, and in the mapping of signal transduction pathways (e.g., RTK-interacting proteins) for RTKs and tyrosine kinase-associated receptors (Figure 2). BRET possesses various advantages compared to standard protein investigation procedures that require invasive or cell-destructive processes such as co-immunoprecipitation or even the previously developed FRET technique. The advances made with BRET, i.e., removing the need for external energy stimulation, have also resulted in an overall improved signal-to noise-ratio when compared to earlier versions of the resonance energy transfer technologies. With respect to cell signaling research, its utility has now significantly gone beyond studying GPCRs. The use of BRET for studying RTKs has great benefit especially as researchers continuously strive to maximize the capacity of BRET as a facilitator to probe for novel drugs and related signaling pathways. In the future, we will most likely witness an increasingly successful number of applications and improvements to the technology.

## Conflict of Interest Statement

The authors declare that the research was conducted in the absence of any commercial or financial relationships that could be construed as a potential conflict of interest.

## Abbreviations

Å, Angstrom; AD, Alzheimer’s disease; APP, amyloid precursor protein; α(v)β(3), alpha(v)beta(3) (integrins); β_2_AR, β_2_-adrenergic receptor; BDNF, brain-derived neurotrophic factor; BRET, bioluminescence resonance energy transfer; cAMP, cyclic adenosine monophosphate; COPD, chronic obstructive pulmonary disorder; EGF, epidermal growth factor; EGFR, epidermal growth factor receptor; ErbB4, erythroblastic leukemia viral (v-erb-b) oncogene homolog 4; EYFP, enhanced yellow fluorescent protein; FAK, focal adhesion kinase; FGF, fibroblast growth factor; FGFR, fibroblast growth factor receptor; FRET, fluorescence resonance energy transfer; GFP, green fluorescent protein; GFP/YFP, green fluorescent protein/yellow fluorescent protein; GHR, growth hormone receptor; GPCR, G protein-coupled receptor; Grb, growth factor receptor-bound protein; HCS, high-content screening; HD, Huntington’s disease; HRG-β1, heregulin beta1; IGF, insulin-like growth factor; IGF-1R, insulin-like growth factor-1 receptor; IGFR, insulin-like growth factor; IL-1β, Interleukin-1 beta; IR, insulin receptor; IRA, insulin receptor alpha subunit; IRB, insulin receptor beta subunit; IRS, insulin receptor substrate; Jak, Janus kinase; Jak/STAT, Janus kinases/signal transducers and activators of transcription; Kit, kit receptor; NGF, nerve growth factor; NT-3, neurotrophin-3; OB-R, leptin receptor; p75NTR, p75 neurotrophin receptor; p85, PI3Kinase 85kd subunit; PDGF, platelet-derived growth factor; PDGF-BB, platelet-derived growth factor beta polypeptide; PDGFR, platelet-derived growth factor receptor; PDGFRA, platelet-derived growth factor receptor, alpha polypeptide; PDGFRB, platelet-derived growth factor receptor, beta polypeptide; PIP3, phosphatidylinositol-3 phosphate; PKA, protein kinase A; PLCγ1, phospholipase C gamma 1; PTB, phosphotyrosine binding; PTP1B, protein tyrosine phosphatase 1B; Pyk2, proline-rich tyrosine kinase 2; Rluc, Renilla luciferase; RTK, receptor tyrosine kinase; SCF, stem cell factor; SH2, Src-homology 2; Shc, adaptor protein 46; Socs, silencers of cytokine signaling; STAT, signal transducer and activator of transcription; Stat5a, signal transducer and activator of transcription 5a; Trk, tropomyosin-related kinase; VEGF, vascular endothelial growth factor; VEGF-C, vascular endothelial growth factor-C; VEGFR, vascular endothelial growth factor receptor.
